# Effects of both climate change and human water demand on a highly threatened damselfly

**DOI:** 10.1038/s41598-021-86383-z

**Published:** 2021-04-08

**Authors:** Rassim Khelifa, Hayat Mahdjoub, Affef Baaloudj, Robert A. Cannings, Michael J. Samways

**Affiliations:** 1 Department of Zoology, University of British Columbia, Vancouver, BC V6T 1Z4 Canada; 2 Biodiversity Research Centre, University of British Columbia, Vancouver, BC V6T 1Z4 Canada; 3 Laboratory LBEE: Biology, Water and Environment, Faculty SNV-STU, University 8 May 1945 Guelma, BP 401 24000, Guelma, Algeria; 4 Royal British Columbia Museum, 675 Belleville Street, Victoria, BC V8W 9W2 Canada; 5 Department of Conservation Ecology and Entomology, Stellenbosch University, Victoria Street, Stellenbosch, 7602 South Africa

**Keywords:** Climate-change ecology, Conservation biology, Freshwater ecology, Ecology, Environmental sciences

## Abstract

While climate change severely affects some aquatic ecosystems, it may also interact with anthropogenic factors and exacerbate their impact. In dry climates, dams can cause hydrological drought during dry periods following a great reduction in dam water discharge. However, impact of these severe hydrological droughts on lotic fauna is poorly documented, despite climate change expected to increase drought duration and intensity. We document here how dam water discharge was affected by climate variability during 2011–2018 in a highly modified watershed in northeastern Algeria, and how an endemic endangered lotic damselfly, *Calopteryx exul* Selys, 1853 (Odonata: Calopterygidae), responded to hydrological drought episodes. Analysis was based on a compilation of data on climate (temperature, precipitation, and drought index), water dam management (water depth and discharge volume and frequency), survey data on *C. exul* occurrence, and capture–mark–recapture (CMR) of adults. The study period was characterized by a severe drought between 2014 and 2017, which led to a lowering of dam water depth and reduction of discharge into the river, with associated changes in water chemistry, particularly during 2017 and 2018. These events could have led to the extirpation of several populations of *C. exul* in the Seybouse River (Algeria). CMR surveys showed that the species was sensitive to water depth fluctuations, avoiding low and high water levels (drought and flooding). The study shows that climate change interacts with human water requirements and affects river flow regimes, water chemistry and aquatic fauna. As drought events are likely to increase in the future, the current study highlights the need for urgent new management plans for lotic habitats to maintain this species and possible others.

## Introduction

The Mediterranean region is a hotspot of biodiversity characterized by a warm, dry, and rapidly fluctuating climate^1,2^. This region is particularly threatened by a rapid increase in both the intensity and length of heatwaves and drought periods^3^. North Africa, in particular, is severely affected by drought^4^, threatening the persistence of wetland integrity and freshwater biodiversity^5^. Although local biodiversity is adapted to cope with drought and heatwave episodes^6^, it is unclear whether species plasticity and adaptive mechanisms are effective enough to cope with the rapid rate of environmental fluctuations^7,8^. In addition, species that depend on permanent water such as many lotic species are most likely more at risk of escaping habitat loss^9,10^ and so require particular attention.

Free-flowing rivers are characterized by a gradient in physicochemical conditions that govern a biotic gradient of vertebrates and invertebrates from upstream to downstream^11^. However, most rivers worldwide are disturbed by dams, which have consequences for hydrology^12^, sediment loads^13^, fish and invertebrate migration^14^, genetic diversity of populations^15^, and other ecological processes^16^. The combined effects of river damming and drought, which is regular in a hot climate^17^, might exacerbate the ecological impacts of climate change and may lead to the extinction of aquatic fauna^18^. For instance, in southwestern Portugal, where climate is warm and dry, the construction of the Alcántara dam caused an increase in the duration and magnitude of the drought in the Tagus basin downstream^17^ with potential consequences for the riparian woody vegetation^19^ and fish communities^20^.

In North Africa, there are both temporary and permanent rivers. In dry years, permanent rivers can undergo a great decline in water level and flow with changes in physical and chemical aspects of the riverine habitat. Given that lotic macroinvertebrate communities are highly dependent on the riverine system^21–23^, the ecological changes related to drought can strongly shape species composition and abundance^24^. However, riverine macroinvertebrates are also affected by other anthropogenic factors^25,26^, particularly land use, pollution, and water extraction for irrigation, which independently or jointly change the structural integrity of watercourses, as well as the physicochemical characteristics of the ecosystem^27–31^. An understanding of how drought and other anthropogenic factors affect populations of aquatic macroinvertebrates is essential to predict changes in biotic communities in the lotic environment. Such information is particularly important because an increase in the frequency and severity of extreme drought events^32^ as well as in anthropogenic factors due to population growth, are expected.

In Algeria, the dragonfly fauna was extensively investigated in the Seybouse watershed during the past decade^33–35^, revealing considerable diversity and endemism. A total of 45 species has been recorded, including the rare North African endemic *Calopteryx exul* Selys, 1853^35^. Although the species was once widespread in northern Africa, its distribution has undergone a substantial retraction due to anthropogenic activities^36^. It is currently restricted to small isolated populations in northern Morocco, northern Algeria, and northwestern Tunisia. This damselfly had not been recorded in Algeria since 1910^37^, and different populations were only discovered after 2007^34^. More recently, a new population was discovered in the northcentral of Algeria^38^. Although the species is currently listed as Endangered on the IUCN Red List^36^, there has not been a substantial conservation effort to manage its habitat or maintain local populations, with several subpopulations in the Seybouse watershed having been lost in the past decade^34^. While anthropogenic habitat degradation is a crucial factor that might have affected the persistence of populations, severe drought could also greatly affect lotic animals. Here, we assess how the extreme drought (2014–2017) affected the distribution of the species in the Seybouse River.

**Figure 1:**
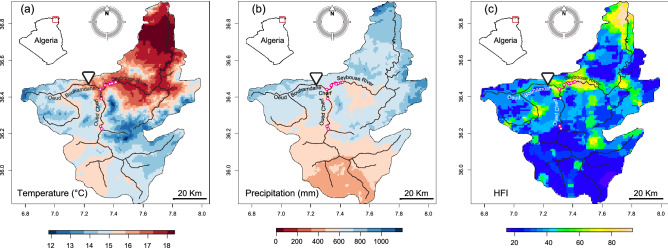
Some environmental characteristics and geographic location of the historical records of *Calopteryx exul* populations in the Seybouse watershed, Northeast Algeria. (**A**) Average annual temperature, (**B**) annual precipitation, and (**C**) human footprint index (HFI). Temperature and precipitation data represent an average across 30 years (1970–2000) and were obtained from WorldClim v2^77^. HFI ranges from 0 (low human influence) to 100 (very high human influence)^79^. Red circles are historical records of *C. exul*. Inverted triangle indicates the Bouhamdane dam. The maps were generated using the software R 4.0.2 (https://www.r-project.org/) and the package raster (https://rspatial.org/raster).

As drought can affect water level, velocity, as well as host-plants used for oviposition by damselflies. Long periods of drought during the reproductive season of *C. exul* may lead to a series of extirpations at different sites. Here we assess the potential impact of hydrological dynamics on *C. exul* distribution by combining historical data of dam water management, survey data of the species’ distribution, daily abundance estimates of adults during the flight period, and capture–mark–recapture of adults. Specifically, we investigate the potential change in the intensity of drought using historical climate data and drought index, estimate the temporal pattern of dam water levels and discharges, and track population persistence and extinction in the Seybouse River of northeastern Algeria (Fig. 1). We also estimated adult abundance and recapture rates of males and females under different water-level conditions. The study uses different aspects of the data to shed light on the potential interaction between climate change and dam water management on *C. exul* population persistence in lotic habitats.

## Results

### Climate variability

**Figure 2:**
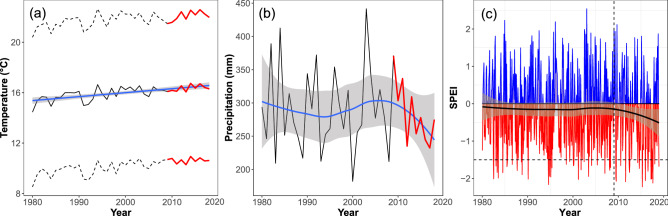
Variability in climatic conditions during 1980–2018 in the Seybouse watershed, northeastern Algeria. (**a**) Temporal pattern of the annual average (solid line), minimum (lower dashed line), and maximum (upper dashed line) temperature. The last ten years are highlighted in red (2009–2018). The blue line is the linear regression and the grey ribbon is the standard error. (**b**) Temporal pattern of the annual precipitation. The last ten years are highlighted in red (2009–2018). The blue line is a loess regression (grey ribbon is the standard error), indicating a decline in the last years. (**c**) Monthly values of Standardised Precipitation Evapotranspiration Index (SPEI) for the study region. Positive values (wet period) are in blue and negative values (dry periods) are in red. The dashed horizontal line is set to − 1.5, indicating severe drought. The dashed vertical line indicates the start of the last decade (2009). The black line is a loess regression, indicating a decline in SPEI during the last decade (a drier period).

The average annual temperature (Tm) across the Seybouse watershed showed a significant increase during 1980–2018, with a temporal slope of 0.03 °C year^−1^ (LM: t = 5.64, P < 0.0001, R^2^ = 0.46) (Fig. 2a). During the decade 2009–2018, increase in Tm was 0.04 °C year^−1^, but it was not significant (t = 1.58, P = 0.15, R^2^ = 0.23). The analysis of the annual temperature extremes (minimum [Tmin] and maximum [Tmax]) for the same decade showed that Tmin did not change significantly (slope = 0.004 °C year^−1^, t = 0.17, P = 0.86, R^2^ ~ 0.00), whereas Tmax showed a marginal increase (slope = 0.07 °C year^−1^, t = 2.11, P = 0.06, R^2^ = 0.28). Annual precipitation across the Seybouse watershed showed high year-to-year fluctuations but no overall significant pattern during 1980–2018 (t = − 0.51, P = 0.61, R^2^ ~ 0.00) (Fig. 2b). However, between 2009 and 2018 there was a temporal decline of annual precipitation of − 10.5 mm year^−1^ (t = − 2.75, P = 0.02, R^2^ = 0.49). Figure 2c shows one-month SPEI values for 1980–2018. Overall, a decline in SPEI was observed in recent years (2009–2018). There were more negative than positive values of SPEI, indicating more frequent dry than wet periods. While only seven values below the threshold of − 1.5 (indicating severe drought) were recorded during 2009–2013 (mean − 1.83 ± 0.20, range − 2.08 to − 1.61, N = 7), 10 of these values were recorded during 2014–2018 (mean − 1.88 ± 0.23 range − 2.22 to − 1.58, N = 10).

### Dam water management

**Figure 3:**
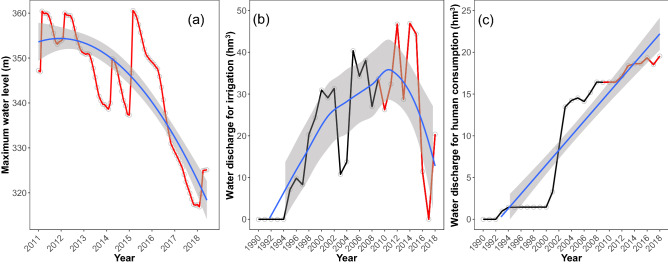
Temporal pattern of water management at the Bouhamdane Dam (Guelma, Algeria). (**a**) Monthly values of the maximum water level during 2011–2018. The blue line is a quadratic regression and the grey ribbon is the standard error. (**b**) Estimated discharge quantity for irrigation during 1990–2018. The blue line is a loess regression and the grey ribbon is the standard error. The last ten years are highlighted in red (2009–2018). (**c**) Estimated discharge quantity for human consumption during 1990–2018. The blue line is a linear regression and grey ribbon is the standard error. The last ten years are highlighted in red (2009–2018).

Dam maximum water level showed a temporal decline between 2011 and 2018 (Fig. 3a), following a quadratic pattern (LM: quadratic effect, t = − 4.92, P < 0.0001; Table S1). The rapid decline started in 2016 when the water depth declined by 9.8% from March 2016 (360.6 m) to June 2018 (325.1 m). Although the water discharge for irrigation decreased in recent years compared to previous decades (LM: quadratic effect, t = − 4.02, P = 0.0004) (Fig. 3b), discharges for human consumption increased continuously (LM: slope = 0.37 hm^3^ year^−1^, t = 7.02, P = 0.0001), albeit at a slower rate in the last decade (Fig. 3c).

The water discharge for irrigation were cyclic events occurring during spring, summer, and autumn (Fig. S1); a period during which the species emerged and reproduced. On average (2011–2018), the largest quantities of releases were recorded in July (6.83 ± 2.87 hm^3^) and August (5.89 ± 2.82 hm^3^), while intermediate quantities were released in late spring (May: 3.54 ± 3.64 hm^3^), early summer (June: 4.65 ± 3.23 hm^3^), and autumn (September: 4.46 ± 2.83 hm^3^, October: 2.83 ± 2.32 hm^3^). Between 2011 and 2016, the peak monthly release ranged from 7.2 hm^3^ in July 2010 to 9.1 hm^3^ in June 2016, and in 2017, there was a small discharge in May (0.26 hm^3^) and June (1.74 hm^3^), and no discharges in 2018, leading to a prolonged drought in the Seybouse River.

### Chemical parameters

To determine whether the physicochemical characteristics of the raw water before treatment showed a temporal pattern during the drought period, 16 environmental variables of the river water (incoming to the Bouhamdane Dam) were assessed during 2012–2018 (Fig. S2). Three parameters (TAC, HCO_3_^−^, and PO_4_^3−^) showed a significant increase (P < 0.05). Four (TH, Ca^2+^, Fe^2+^, and NH_4_^+^) showed a marginally significant increase (P = 0.05–0.07), whereas the other variables did not show a significant pattern, albeit most of the non-significant increases (e.g. Conductivity, Mg^2+^, Cl^−^, and RS, P = 0.12–0.16) were probably due to the low sample size (7 years of data).

### Damselfly distribution response

**Figure 4:**
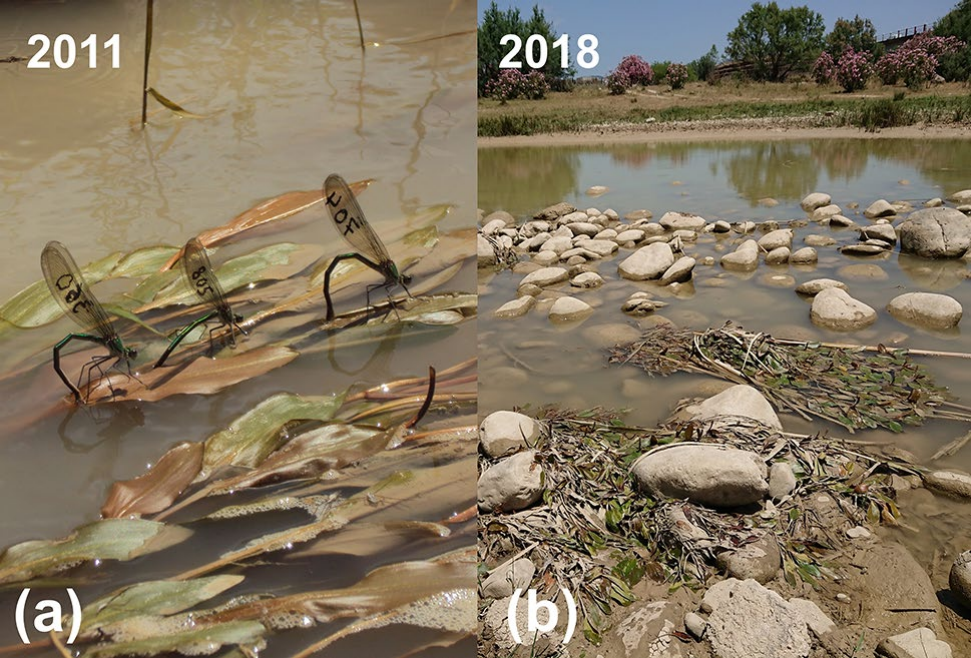
Reproduction of *Calopteryx exul* in the Seybouse River in 2011 (**a**) and a huge drop in water level of the river in 2018. Note that the patches of long-leaf pondweed (*Potamogeton nodosus*), a preferred oviposition site for the species, which typically float on the water surface are dry due to water abstraction (Credit: Rassim Khelifa).

The largest number of subpopulations of *C. exul* in the Seybouse River was recorded in 2011 (N = 8; Table S2, Fig. S3a, b). Of these populations, three were permanent subpopulations during 2011–2016, whereas four were transitory (disappeared and reappeared). In 2018, the number of subpopulations in the river declined to one transitory subpopulation, although previously extirpated since 2012 (Fig. S3c). All three permanent subpopulations, where reproduction commonly occurred, disappeared in 2018 (Fig. 4a,b). Thus, during the period 2011–2018, there was a loss of 0.85 subpopulations per year.

A combination of all sites from the entire watershed shows that *C. exul* lived in areas where the average human footprint index (HFI: a metric that varies between 0 and 100 and indicates human influence) within 2 km-radius hexagon was 55.5 ± 14.9 with a minimum of 23.4 and a maximum of 75.6. This value corresponds to the 83 percentile of the HFI occurring in the main watercourses of the watershed which had a mean HFI of 38.2 ± 17.4 (range 21–93).

### Adult abundance and recapture rate

**Figure 5:**
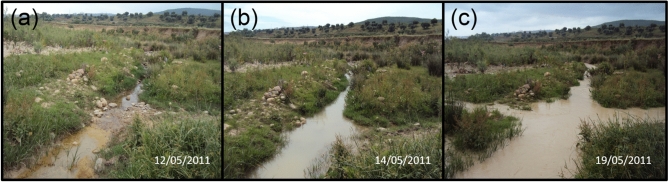
Variability of stream water level where the endangered *Calopteryx exul* reproduced, Seybouse River, northeastern Algeria. (**a**) stream nearly dry, (**b**) intermediate water level (preferred level of the damselfly), and (**c**) flooded stream. Note that the three states of the stream were recorded within a single week (Credit: Rassim Khelifa).

**Figure 6:**
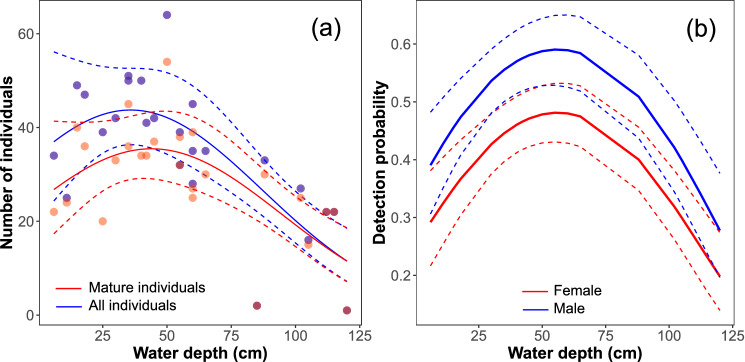
The effect of stream water depth on the number of adult individuals (**a**) and the recapture probability of marked male (blue) and female adults (red) of *Calopteryx exul* (**b**). (**a**) The fitted line is a negative binomial regression for all individuals (blue) and mature individuals only (red). (**b**) The fitted line is a prediction of a Cormack–Jolly–Seber model for capture–mark–recapture including a constant survival.

Since the water level of the reproductive sites of *C. exul* fluctuated regularly within a season (Fig. 5), mainly due to dam water discharges and water pumping for irrigation, we investigated the effect of water-level fluctuation on the abundance and detection (recapture) of the species. Based on the temporal pattern of daily abundance in the reproductive season of 2011 at El Fedjoudj P, the number of adult (all and only mature individuals) *C. exul* showed a quadratic pattern with water depth (Table S3). The largest number of adult *C. exul* individuals was recorded on days when water depth was intermediate (0.2–0.7 m), while a relatively lower number was noted on days with low water depth (< 20 cm), and lowest numbers were recorded during floods (> 1 m) (Fig. 6a). A great reduction (drought) or increase (flooding) in water level resulted in the disappearance of the preferred reproductive substrates. Drought and subsequent water level drop resulted in bank vegetation being far from the water, while in turn, flooding led to submergence of the oviposition host plants. Even if a number of individuals was recorded during drought, they were not showing reproductive behavior.

Our analysis included 313 marked adults, of which 56.8% were recaptured at least once. The average ± SD number of recaptures was 2.78 ± 2.46 with a median of 2 and a range of 1–19 (Fig. S4). Capture–mark–recapture data showed that the best model for the recapture probability was a quadratic effect of water depth and sex, with lowest recapture probabilities recorded in days with very low or very high water levels, whereas the highest probabilities were recorded at intermediate water levels (Fig. 6b; Table S4). Given our sampling was extensive, the absence of both males and females indicated a potential dispersal out of the sampling area due to hydrological changes. Males had a larger recapture probability than females.

## Discussion

Our study showed that climate change could have an interactive effect with dam water management, exacerbating drought intensity, and ultimately affecting aquatic organisms. Specifically, the results showed that the prolonged severe drought changed water chemistry, including an uptake in the level of pollutants, and reduced the volume of dam water discharge released into the river yet maintaining water discharges for human consumption. This greatly affected the river water level and flow, and consequently, the distribution of the endangered North African endemic damselfly, *C. exul*. We highlight the need for action plans that account for climate change scenarios, dam water management plans, wetland bathymetry and hydrology, irrigation requirements, and human disturbance^39,40^ to determine the future impacts of climate change on freshwater ecosystems and for managing biodiversity effectively.

### Climate change and dam water management

North Africa is one of the driest and warmest regions in the world, which has also undergone severe climate change^4^. Our results showed that the thermal and hydric conditions fluctuated during 2009–2018, showing warmer conditions and more severe drought in later years of the study period. In this region, most freshwater ecosystems are present in the coastal area where communities are limited in the south by the Sahara Desert, and to the north by the Mediterranean Sea, limiting the extent of potential refuges for species. This means that particular attention should be given to the impact of climate change on freshwater communities^41,42^ where species physiology, behavior, and distribution are particularly sensitive to environmental changes^41,42^. Increased temperatures, together with reduced precipitation and changes in the physicochemical properties of the water^43^ might have induced physiological stress^44^, reduced physical space, while also increasing vulnerability to competition and predation^45^, and weakened habitat connectivity^46^ which under extreme conditions may lead to drought and local extirpations^47^. Here, we show the consequences of the combined effect of anthropogenic control of river water flow and climate change, leading to increased magnitude and duration of drought, which is expected to increase in frequency in the following years due to climate change^17^.

The results show that the water discharges declined in 2017, stopping completely in 2018 after extreme drought events, leading to a huge reduction in river water flow (river width and water depth). The decision to stop water discharges was the result of a great decline in the dam water supply for human consumption. The river was further exploited for agriculture with water pumps, leading to a complete drought in some parts of the river. This reduction in water quantity and a constant input of agricultural run-off, sewage and industrial discharges reduced water quality for aquatic fauna, crops, and livestock^48^. The predicted increase in water consumption due to human population growth and irrigation is likely to intensify hydrological drought in North Africa^49^. Future models should include all these components to predict future climate effects on the local lotic fauna^50^.

### Water physicochemical properties

The temporal pattern of water chemistry showed that many parameters, including indicators of pollution, increased during the drought event. Phosphate increased substantially to levels that foster eutrophication (> 0.1 mg/L) and other adverse environmental effects^51^. Ammonia, which is toxic to aquatic fauna at high concentrations^52^, has also increased in recent years. These changes in water chemistry are probably due to drought which reduces water levels and increases the concentration of nutrients and toxic compounds in the water. The data were used only to infer potential temporal changes in water chemistry and quality in the dam, rather than the levels encountered downstream where *C. exul* occurs. For instance, phosphate, ammonia, and sulfate levels recorded in raw water before treatment in 2013 were > 60, ~ 50, and ~ 3 times lower than those recorded at Medjez Amar (Seybouse River, upstream) in the same year^53^, respectively. Such increases are due to sewage from high human populations, intensive land use by agriculture, and industrial pollution. Therefore, drought not only affects the physical aspects of the water but also its chemistry, making the environmental conditions of lotic habitats very stressful and pushing the aquatic fauna beyond their physiological tolerance.

### Impacts on *Calopteryx exul* populations

The severe drought of 2014–2017 has probably caused directly and/or indirectly the extirpation of different populations of the endangered damselfly *C. exul* because it coincided with the rapid loss of populations. The distribution of *C. exul* in the Seybouse River has rapidly declined over the past decade^34^. Its rapid local extirpation rate in the region now suggests that the species should locally be raised to the category of Critically Endangered in accordance with IUCN criteria B2 and E, which take into account the range size, severity of habitat fragmentation, and local extinction probability over the decade. The current study shows that subpopulations that have shown the most resilience in the past decade^34^ were extirpated in 2018, and the remaining populations colonized sites that have experienced extirpation in the past. It is important to point out that the species could have found refuges elsewhere in the watershed, which warrants further investigations in future monitoring. Nevertheless, the future persistence of the species in the Seybouse River is threatened by future drought events, as well as by dam water management for human needs.

Since *C. exul* is highly dependent on flowing water during its entire life cycle, drought per se (excluding other interactive effects) could have fitness consequences for all life stages. First, eggs are typically laid inside floating leaves near the bank of the watercourse^34^. The reduction in water levels in dry conditions causes desiccation of the plant and eventual mortality of eggs. Second, larvae that typically occur in relatively fast-flowing water are also highly affected by drought as the water velocity and physical aquatic space decline. Although larvae can find refuge in humid areas, a prolonged drought could cause mortality due to predation or desiccation and other non-lethal effects (depleted energy reserves and reduced body size). Third, even if adults are adapted to disperse in the case of drought, and seek more suitable sites to reproduce or hospitable refuge sites to retreat to but not reproduce^54^, drought can have great consequences for the species’ population dynamics. The life span of mature adults is short (< 1 month), and so the species is in a ‘race against time’, where every day of the reproductive season counts in the overall population dynamics of the species. During an extended drought, suitable reproductive habitats become scarce or even disappear, with females laying only a small fraction of their clutch, if any at all, before they die. This affects the initial population size (number of viable eggs) and, consequently, the size of the emerging population in the next year or the second emergence season of the same year^55^.

Unfortunately, the reproductive season of *C. exul* overlaps with both the dry season and the increased need for agricultural water pumping. The Seybouse River is bordered by agricultural lands of various crops and fruits, some of which are very demanding on water supply (e.g. watermelons, melons, and tomatoes). This means that there is an already existing high water use for irrigation, which reduces water levels of the river and streams. In dry years, water quantity in the river is reduced but the water pumping demands remain constant, which increases the effects on hydrology and causes cascading effects on aquatic and terrestrial communities^56^. After an extremely dry period and a major decline in dam water quantity threatening water security, the dam stops all water discharges and the river is exploited by farmers until it becomes completely dry. While Bouhamdane Dam occurs in one of the tributaries of Seybouse River, the other main tributary (Cherf River) also has a dam (Foum El-Khanga Dam) with a capacity of 157 million m^3^. This dam is 15 km away from the remaining *C. exul* population of the Cherf River near Ain Makhlouf, which exists in a highly degraded site due to the construction of a bridge. Therefore, all known subpopulations of the Seybouse watershed are threatened by the combined effect of dam and drought.

Besides drought, flooding due to dam water discharges changes the water flow dynamics and pushes *C. exul* to the other extreme of inhospitable hydrological conditions. Flooding may drift larvae to unsuitable habitats where food availability and water quality are low and mortality risks are high^57,58^. Flooding often changes the community structure of the bank vegetation and reduces the availability of oviposition sites. Because the species is selective of habitat characteristics such as water flow and bank vegetation^33^, males do not show reproductive behavior (territoriality) and females stop oviposition during high water, because water velocity exceeds the optimal conditions. Future models for the prediction of the population dynamics of the species should take into account not only drought but also flooding events as important factors that influence demographic parameters of all life stages.

### Capture–mark–recapture

Further evidence that the species is sensitive to hydrological regimes is shown by analysis of capture–mark–recapture data. There is a noticeable relationship between the recapture probability of the adults and stream water depth. In both sexes, the recapture rate declined greatly when the water levels were very low (drought) or very high (flooding). This suggests that the species prefers intermediate water depth (also correlated with intermediate water velocity), probably because it confers the optimal conditions for oviposition and survival of eggs and larvae^59^. In non-optimal conditions, *C. exul* appears to disperse in search of other potential sites, and to escape adverse local environmental change, similar to other species of Calopterygidae^60–62^. Previous studies on the species have shown that habitat improvement (due to increased availability of reproductive territories) increases the recapture probability, the local population size of the adults, and the number of reproductive events^34,63^. The sexual difference in the average recapture rate of *C. exul* is common in odonates, and it is due to behavioral differences where males are territorial, more conspicuous, and permanently near the water, whereas females are more cryptic and may leave the water after oviposition^64^. Nevertheless, both sexes responded similarly to variation in stream levels due to dam water management. It is unclear whether the new patches colonized after habitat quality degradation (drought or flooding) are only temporarily suitable for reproduction or whether the deposited eggs will survive to emergence.

### Management perspectives

Drought is a common event in the Mediterranean climate, with future increases in both frequency and duration imminent^65^. Severe drought could exacerbate the impact of other disturbances such as pollution and water abstraction for irrigation. Effective measures are required for the management of water supplies for consumption and irrigation in conjunction with the conservation of vertebrates and invertebrates of the rivers^66^. We discuss a suite of actions that could improve the conservation status of *C. exul* and increase the resilience of lotic biodiversity against extreme weather events.

The creation of an artificial ditch system that could be used both for irrigation and refuge for the lotic fauna in case of an extreme drought could attenuate the impact of drought on lotic ecosystems^67^. In South Africa, some Cape endemic species occupy artificial reservoirs during severe drought events and use them as refuges until the favorable conditions return in their reproductive areas^68^. This escape behavior is typical of drought-adapted lotic endemics that are dependent on perennial rivers and streams to reproduce and maintain viable populations. Ideally, a network of artificial watercourses at the watershed scale (conservation corridors) allows the species to disperse, escape drought, and potentially find suitable patches to establish populations^69^. The success of such artificial sites will likely increase if these sites support some attractive habitat elements such as bank vegetation for perching, and floating leaves to guard oviposition sites. For instance, *C. exul* adults could be lured to specific areas by providing oviposition sites (floating leaves of plants)^34,63^, which could be displaced back to their original sites when the water level return to normal.

In North Africa, lotic habitats receive much less conservation attention than do lentic habitats. Recognizing the ecological and socioeconomic importance of lotic ecosystems is an essential goal for creating protected areas in local watersheds^70^. In the case of *C. exul*, and in view of the loss of several of its subpopulations these last few years, one approach to save it from extinction would be to designate it a protected area where there is a reduced riparian disturbance. Such habitat protection is likely also to have an umbrella effect, benefitting other threatened endemic dragonflies, such as *Gomphus lucasii* Selys, 1849, as well as a wide spectrum of invertebrates and vertebrates.

While *C. exul* might have some resilience to drought, the pumping of water from the river and streams is a supplementary pressure that could push the species to the limits of its resilience by extending the drought period and adversely affecting the larvae out of the water. Watercourse modification is another element that changes water velocity and modifies the integrity of the habitat for aquatic macroinvertebrates in general^71^. Strict regulations are required to limit the amount of water pumped out of natural habitats. Furthermore, alternative water sources for irrigation, such as artificial ditch systems that direct dam water appropriately, are key to solving the problem.

Given that most of the watershed is occupied by agricultural lands, establishing new conservation and restoration measures that promote eco-friendly farming practices and more natural landscape configuration should be at the forefront of the strategic conservation plan^72^. Shifting farming practices that rely heavily on fertilizers and pesticides to more organic farming will most likely reduce the toxic run-off into rivers and streams, improve water quality at reproductive sites, ameliorate habitat quality in foraging sites, and improve food quality (uncontaminated prey)^73,74^. Since the agricultural lands in the watershed are highly simplified monocultures with very low habitat complexity, the addition of habitat elements such as trees, shrubs, and mixture of crop species alongside native grasslands will improve the landscape complexity, maintain biodiversity, and increase ecosystem resilience^75^. Such a shift to diversified agroecosystems should be implemented gradually following awareness-raising among the public and other stakeholders, and subsequently policy directions.

Instigation of a regular long-term monitoring program at the watershed scale would be crucial for the assessment of population trends, species sensitivity to environmental and anthropogenic perturbation, and the success of newly implemented management plans. In addition, even if conservation authorities will not devote much attention and resources to watershed conservation, a well-planned community science initiative could play a considerable role in data collection, public education, and conservation advocacy. Ultimately, effective management of riverine ecosystems as well as future climate impacts requires a multidisciplinary collaborative network involving scientists from a variety of disciplines—ecology, climatology, hydrology, sociology, economics. Unfortunately, we are currently far from having such an established network along the Seybouse River, despite the urgency of the situation.

## Methods

### Study site

The study was conducted in the Seybouse River, located in northeastern Algeria (Fig. 1). The river flows into the Mediterranean Sea and is formed by the confluence of oued Bouhamdane and oued Cherf at Medjez Amar, 10 km west of Guelma city.

The Bouhamdane Dam is located in the Hammam Debagh province, 23 km west of Guelma, Algeria (36°27′40″N, 7°14′15″E). The dam is on the Bouhamdame River 7 km upstream from the origin of the Seybouse River (36°26′35″N, 7°18′39″E) and restricts the flow of the Bouhamdame River, which feeds into the Seybouse River. The surface area of the dam is 1070 km^2^ with a maximum water level of 370 m. It has a capacity of 220 hm^3^ and a velocity of 2240 m^3^ s^−1^. It provides drinking water for different provinces (Guelma, Ben Djerrah, Medjez Amar, Hammam Debagh, and Ain Hessainia) which require an estimated 9 hm^3^ year^−1^^76^. In this region, the wet season (mild moist winters) spans October to April/May, and the dry season (hot and dry summers) June to September, coinciding with the reproductive season of most aquatic insects, including odonates^35^. Climatic and anthropogenic characteristics of the Seybouse watershed are presented in Fig. 1.

### Climate and dam data

To characterize the regional climatic conditions in the Seybouse watershed, annual average temperature (Tm) and annual precipitation (P) data (averaged across 1970–2000 and the Seybouse watershed) obtained from WorldClim v2 with 2.5-min spatial resolution were used^77^. Historical monthly weather data for 1980–2018^78^ were averaged across years (summed in the case of precipitation) and the Seybouse watershed to assess the temporal trends of Tm and P. To determine the human influence on the watershed and *C. exul* localities, the global human footprint index (HFI) with a spatial resolution of 1 km^2^ was used^79^. HFI is a multivariate metric that includes population density, human land use, infrastructure disturbance (e.g. buildings, night-time artificial lights), and human access (e.g. road, railroads). To evaluate the intensity of human influence in localities where *C. exul* lives, and across the water system of the Seybouse watershed, the average HFI at 4 km^2^-hexagon grids was calculated. One-month values of Standardized Precipitation Evapotranspiration Index (SPEI), which is a metric that measures drought severity (intensity and duration), were obtained for the study region (36.25°N, 7.25°E) from the global SPEI database^80^. One-month SPEI was used (instead of 3- and 6-month periods) to avoid temporal autocorrelation. A threshold value of − 1.5 was used as an indicator of a significant drought^81^. To assess temporal changes in dam water levels and discharges, data on the monthly maximum water level and monthly quantity of water discharge of Bouhamdane Dam for the period of 2011–2018 were acquired from the dam management executives. In addition, to determine whether the physicochemical properties of the water changed during the drought period, we tracked temporal trends in water physicochemical characteristics of the raw incoming water to the Bouhamdane Dam before treatment using the average annual values of 16 parameters surveyed during 2012–2018, namely, pH, turbidity, conductivity, Total alkalinity (TAC), Total hardness (TH), Bicarbonate (HCO_3_^-^), Calcium (Ca^2+^), Magnesium (Mg^2+^), Chloride (Cl^−^), Dry residue (RS), Nitrate (NO_3_^−^), Iron (Fe^2+^), Sulfate (SO_4_^2−^), Phosphate (PO_4_^3−^), Ammonium (NH_4_^+^), Organic matter (MO).

### Sampling methods

Surveys were conducted on the subpopulations of *C. exul* in the reproductive season of 2017 and 2018. The occurrence of adult individuals (teneral, immature, and mature) were recorded using a 200 m-transect along the bank of the water course. In addition, we used species occurrence data from sites as early as 2011, when the number of subpopulations in the Seybouse was the highest^34^. Each subpopulation was visited at least three times during the reproductive season. For this paper, we assess only subpopulations that occur in the Bouhamdane and Seybouse Rivers because the latter are sensitive to Bouhamdane Dam’s water management.

In the reproductive season of 2011, an extensive capture–mark–recapture scheme was carried out in the Seybouse River upstream to estimate population size, assess emergence patterns, describe the reproductive behavior, and understand dispersal habits of the species^35,55,82^. During 28 April–29 May 2011, daily captures of *C. exul* adults were made in a channel of the river (El Fejdoudj P; 36°28′21″N, 7°22′15″E) across a transect of 100 m during the morning (09:00–12:00). After capturing the damselflies with hand nets, individual alphanumeric codes were marked on a wing with a permanent marker. The marking took only a few seconds and the insect was released immediately in the same location. Daily recaptures (resightings) were carried out. The total number of adults (males and females) was also recorded across the transect. Since the species is morphologically and behaviorally conspicuous, it is highly likely that all individuals at the site were recorded. As the water depth of the study site fluctuates from one day to another (Fig. 5), water depth was daily estimated from a specific location at the center of the waterbed of the studied channel using a graded stick. The fluctuation of water level at the center of the waterbed is reflective of the general changes in the hydrology of the watercourse. We tested for the correlation of this hydrological fluctuation with the variation in recapture rate using the capture history of 313 marked individuals. It is important to note that variation in water depth at a specific location is also an indicator of a change in water flow; an important environmental feature that highly influences the likelihood that a *Calopteryx* species reproduces at a given site^59^.

### Statistical analyses

All analyses were carried out using R 3.2.3^83^. The yearly changes in dam water level were analyzed with polynomial regression. Linear regressions were used to assess the yearly changes of water discharges, average, minimum and maximum of annual temperature, and annual precipitation. Capture–mark–recapture data were analyzed using a Cormack–Jolly–Seber (CJS) model with the *RMark* package^84^. The assumptions of the model were checked with the function *release.gof* which provides tests that assess the independence and transience (TEST2, TEST3, and TOTAL)^85^. All tests showed non-significance (P > 0.50), suggesting that the data meet the requirements for the CJS model^86^. Since the recapture probability (p) was the variable of interest, the survival probability (Phi) was fixed at one, and tested for the effect of individual (sex) and environmental covariates (water depth [WD]). Candidate models were selected starting with a simple model including the linear effect of one covariate (sex and water depth) to a more complex model including polynomial terms of the water depth (WD + WD^2^), and the additive effects of the covariates (WD + WD^2^ + sex). The models were first computed, then model selection based on the AICc (corrected Akaike information criterion) was performed. The effect of water depth on the number of adults across the watercourse was analyzed using a negative binomial regression. Values are mean ± SD.

## Data and materials availability

Data were deposited in a GitHub repository: https://github.com/rassimkhelifa/Data.

## Supplementary Information

Supplementary Information.
