# Supplementary material for: Effects of both climate change and human water demand on a highly threatened damselfly
Source: Sci Rep. 2021 Apr 8;11:7725. doi: 10.1038/s41598-021-86383-z (PMC8032742; doi:10.1038/s41598-021-86383-z)
Supplement: Supplementary file 1 — Supplementary Information. [file 41598_2021_86383_MOESM1_ESM.docx]

Supplementary material

**Effects of both climate change and human water demand on a highly threatened damselfly**

Rassim Khelifa^1,2*^, Hayat Mahdjoub^2^, Affef Baaloudj^3^, Robert A. Cannings^4^ and Michael J. Samways^5^

^1^ Department of Zoology, University of British Columbia, Vancouver, Canada, BC, V6T 1Z4

^2^ Biodiversity Research Centre, University of British Columbia, Vancouver, Canada, BC, V6T 1Z4

^3^ Laboratory LBEE: Biology, Water and Environment, Faculty SNV-STU, University 8 May 1945 Guelma, BP 401 24000, Guelma, Algeria

^4^ Entomology, Royal British Columbia Museum, 675 Belleville Street, Victoria, B.C., Canada V8W 9W2

^5^ Department of Conservation Ecology and Entomology, Stellenbosch University, Victoria Street 7602, Stellenbosch, South Africa

*Corresponding author. Email: [rassimkhelifa@gmail.com](mailto:rassimkhelifa@gmail.com)

**Supplementary figures**

**
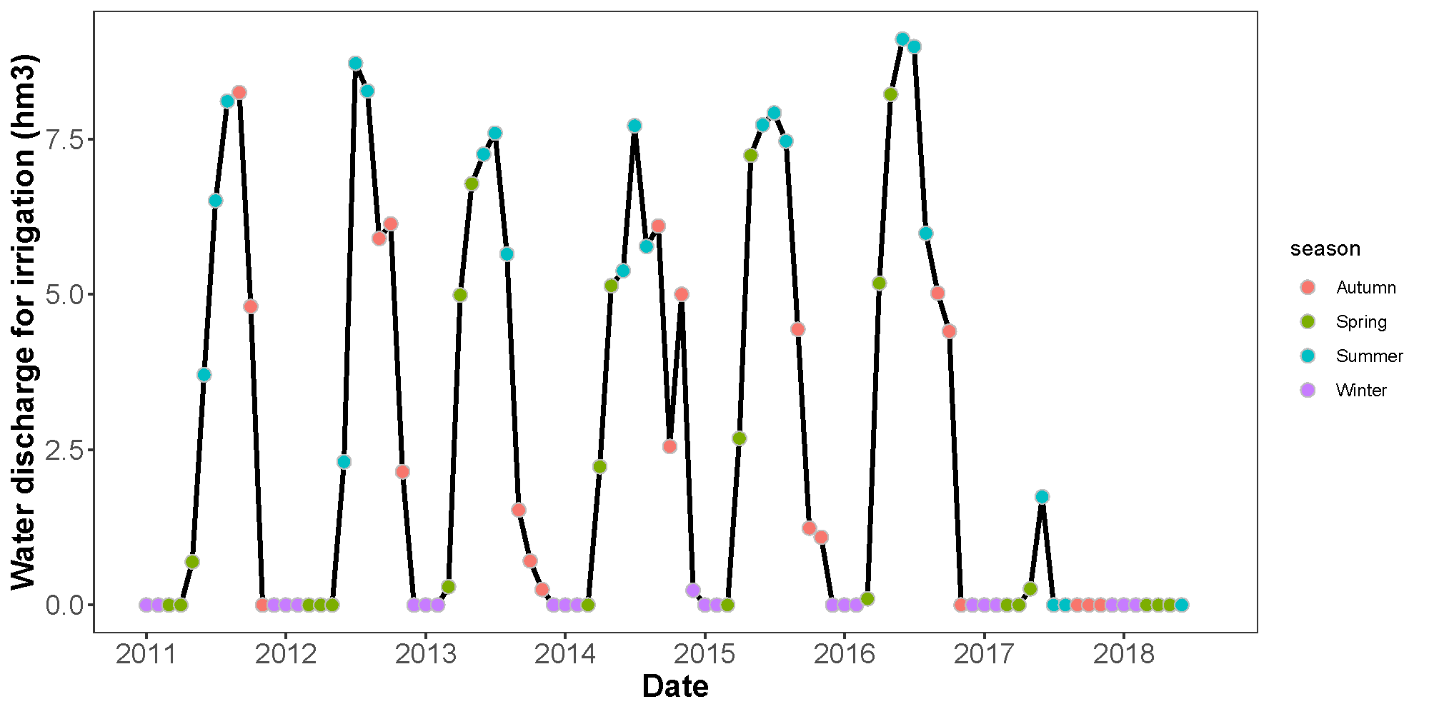
**

**Figure S1.** Temporal pattern of the estimated discharge quantities released for irrigation by the Bouhamdane Dam (Guelma, Algeria) during 2011-2018. Here, autumn refers to September-November, winter to December-February, spring to March-May, and summer to May-July.

**
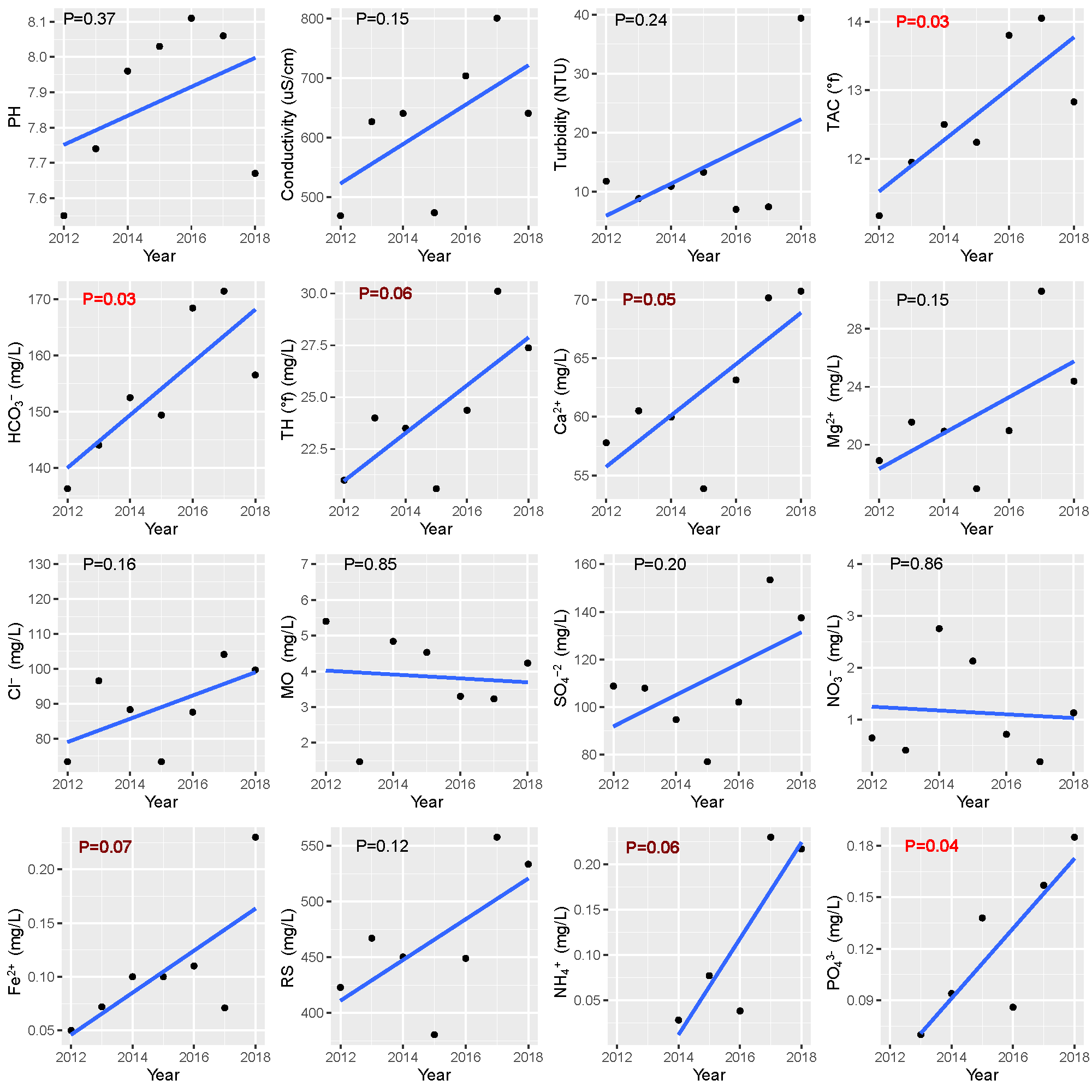
**

**Figure S2.** Temporal pattern of physicochemical characteristics of the raw untreated water during 2012-2018 received by Bouhamdane Dam from the river. Blue lines are linear regressions. P-values are provided for each parameter; significant ones (P<0.05) are indicated in red whereas marginally significant ones (P= 0.05-0.07) are in brownish red. TAC: Total alcalinity, HCO_3_^-^: Bicarbonate, TH: Total hardness, Ca^2+^: Calcium , Mg^2+^: Magnesium , Cl^-^: Chloride , RS: Dry residue, NO_3_^-^: Nitrate , Fe^2+^: Iron, SO_4_^2-^: Sulfate , PO_4_ ^3-^: Phosphate, NH_4_^+^: Ammonium, MO: Organic matter.****

**Figure S3.** Study site showing the temporal change of the number of subpopulations of *Calopteryx exul*. (a) All populations recorded in the Seybouse watershed. The zoomed part of the map includes only the subpopulations that are threatened by the management of the Bouhamdane Dam (blue square). (b) The number of subpopulations recorded in 2011. (c) The number of subpopulations recorded in 2018. The map was created with MapInfo Professional software (version 15.0, http://www.pitneybowes.com/us/location-intelligence/geographic-information-systems/mapinfo-pro.html).

**
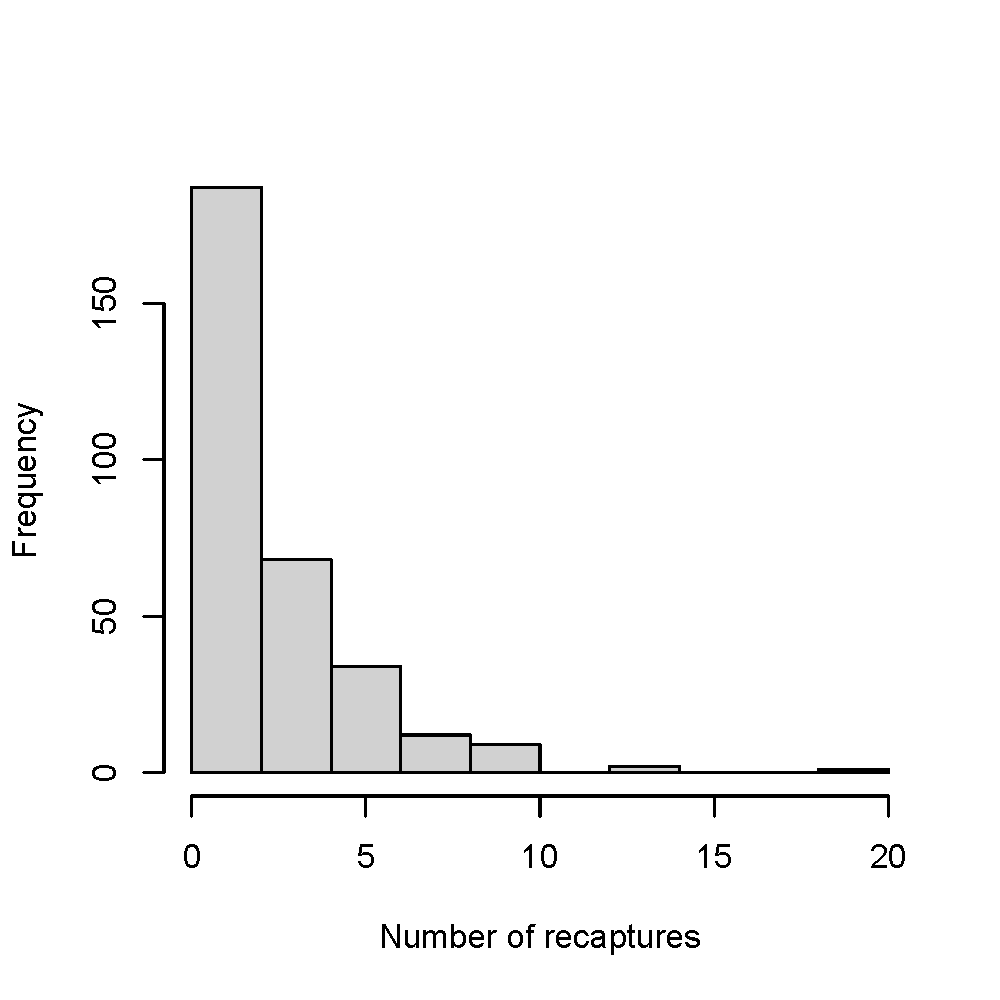
**

**Figure S4.** Frequency of recaptures of marked individual of *Caloptery exul* in the capture-mark-recapture survey in the Seybouse River.

**Supplementary tables**

**Table S1.** Summary statistics of the polynomial regression assessing the temporal pattern of the Bouhamdane Dam maximum water level for the period of 2011-2018. Date was converted to a continuous variable and standardized.

|  | Estimate | Std. Error | t value | Pr(>\|t\|) |
| --- | --- | --- | --- | --- |
| Intercept | 347.38 | 1.09 | 317.341 | < 0.0001 |
| Date | -10.90 | 0.75 | -14.467 | < 0.0001 |
| Date² | -4.25 | 0.86 | -4.929 | < 0.0001 |

**Table S2.** Study sites of *Calopteryx exul* in the Seybouse River where eight subpopulations were surveyed between 2011 and 2018. 1: Presence; 0: absence.

| Site | Latitude (°N) | Longitude (°E) | 2011 | 2012 | 2013 | 2014 | 2015 | 2016 | 2017 | 2018 |
| --- | --- | --- | --- | --- | --- | --- | --- | --- | --- | --- |
| ElFedjoudj Pont | 36.481978 | 7.414653 | 1 | 1 | 1 | 1 | 1 | 1 | 1 | 0 |
| ElFedjoudj S | 36.479519 | 7.405678 | 1 | 1 | 1 | 1 | 1 | 1 | 1 | 0 |
| ElFedjoudj E | 36.475472 | 7.388636 | 1 | 1 | 1 | 1 | 1 | 1 | 1 | 0 |
| ElFedjoudj P | 36.472745 | 7.376172 | 1 | 1 | 1 | 1 | 0 | 0 | 0 | 0 |
| ElFedjoudj H | 36.474812 | 7.374233 | 1 | 1 | 0 | 0 | 0 | 0 | 0 | 0 |
| ElFedjoudj G | 36.476802 | 7.367433 | 1 | 1 | 1 | 1 | 1 | 0 | 0 | 0 |
| Salah Salah 2 | 36.465433 | 7.340964 | 1 | 0 | 0 | 0 | 0 | 0 | 0 | 0 |
| Salah Salah | 36.462089 | 7.339578 | 1 | 0 | 0 | 0 | 0 | 0 | 0 | 1 |
| Total |  |  | **8** | **6** | **5** | **5** | **4** | **3** | **3** | **1** |

**Table S3.** Summary statistics of the negative binomial regression assessing the effect of stream water depth on the number of adult *Calopteryx exul* in the Seybouse River in 2011. (a) All individuals (teneral, immature, and mature); (b) only mature individuals.

| Age cohort | Parameter | Estimate | Std. Error | z value | Pr(>\|z\|) |
| --- | --- | --- | --- | --- | --- |
| (a) All individuals | (Intercept) | 6.252 | 1.979 | 3.159 | 0.00158 |
|  | WD | 0.019 | 0.010 | 1.879 | 0.06025 |
|  | WD² | 0.000 | 0.000 | -2.859 | 0.00425 |
|  | JD | -0.020 | 0.015 | -1.376 | 0.16868 |
|  |  |  |  |  |  |
| (b) Only mature individuals | (Intercept) | 5.647 | 2.072 | 2.725 | 0.00642 |
|  | WD | 0.022 | 0.011 | 2.080 | 0.03754 |
|  | WD² | 0.000 | 0.000 | -2.793 | 0.00523 |
|  | JD | -0.019 | 0.016 | -1.188 | 0.23470 |

WD: water depth; JD: Julian date

**Table S4.** Capture-mark-reapture model selection for recapture probability of *Calopteryx exul* in the Seybouse River in 2011. The best model is in bold.

| Model | npar | AICc | ΔAICc | weight | Deviance |
| --- | --- | --- | --- | --- | --- |
| Phi(.) p(WD+WD²+Sex) | **5** | **2386.7** | **0.000** | **0.979** | **1419.9** |
| Phi(.) p(WD+WD²) | 4 | 2394.3 | 7.663 | 0.021 | 1429.6 |
| Phi(.) p(WD+Sex) | 4 | 2414.1 | 27.399 | 0.000 | 1449.3 |
| Phi(.) p(Sex) | 3 | 2416.4 | 29.713 | 0.000 | 1453.6 |
| Phi(.) p(WD) | 3 | 2419.0 | 32.320 | 0.000 | 1456.2 |
| Phi(.) p(.) | 2 | 2423.8 | 37.094 | 0.000 | 1463.0 |

Dot (.) : parameter set to 1. WD: water depth. npar: the number of parameters.
